# (3-Anilino-1-phenyl­imino­thio­ureato)chloridodimethyl­tin(IV)

**DOI:** 10.1107/S160053680900988X

**Published:** 2009-03-25

**Authors:** Jorge A. Delgado D., Coco K. Y. A. Okio, Richard Welter

**Affiliations:** a Departamento de Quimíca, Universidad Nacional de Colombia, Sede Bogotá, Bogotá, Colombia; b Laboratoire DECOMET, UMR–CNRS 7177, Université Louis Pasteur, 4 rue Blaise Pascal, 67000 Strasbourg, France

## Abstract

In the title compound, [Sn(CH_3_)_2_(C_13_H_11_N_4_S)Cl], the Sn atom is five-coordinated in a distorted trigonal-bipyramidal geometry, with two methyl groups and one S atom in the equatorial plane, and one N atom and the Cl atom occupying the apical positions.

## Related literature

For related structures, see: Labib *et al.* (1996 ▶). For the biological and pharmaceutical applications of organotin derivatives, see: Davies & Smith (1982 ▶); Diop *et al.* (2003 ▶); Okio *et al.* (2003 ▶).
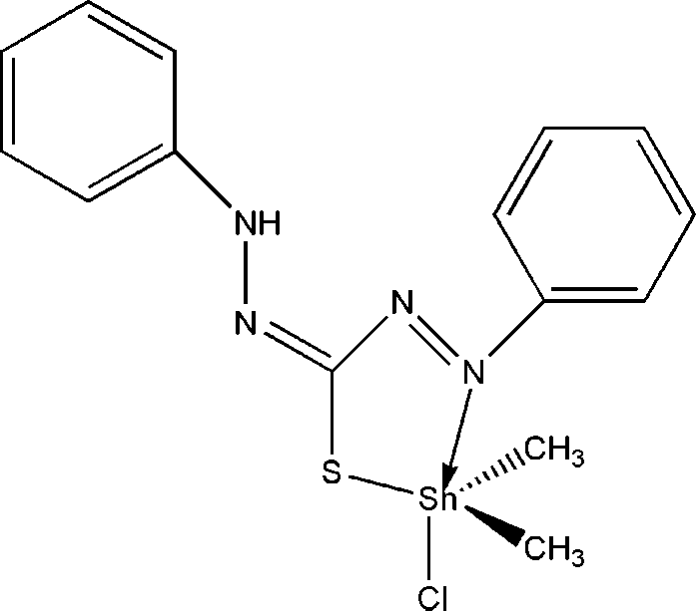


## Experimental

### 

#### Crystal data

[Sn(CH_3_)_2_(C_13_H_11_N_4_S)Cl]
*M* *_r_* = 439.53
Orthorhombic, 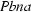

*a* = 11.6850 (2) Å
*b* = 14.8920 (5) Å
*c* = 20.6790 (7) Å
*V* = 3598.42 (18) Å^3^
*Z* = 8
Mo *K*α radiation
μ = 1.69 mm^−1^
*T* = 173 K
0.10 × 0.10 × 0.10 mm

#### Data collection

Nonius KappaCCD diffractometer
Absorption correction: none
25644 measured reflections
4130 independent reflections
2731 reflections with *I* > 2σ(*I*)
*R*_int_ = 0.115

#### Refinement

*R*[*F* ^2^ > 2σ(*F* ^2^)] = 0.044
*wR*(*F* ^2^) = 0.137
*S* = 1.09
4130 reflections
199 parameters
H-atom parameters constrained
Δρ_max_ = 1.13 e Å^−3^
Δρ_min_ = −1.84 e Å^−3^

### 

Data collection: *COLLECT* (Nonius, 1998 ▶); cell refinement: *SCALEPACK* (Otwinowski & Minor, 1997 ▶); data reduction: *DENZO* (Otwinowski & Minor, 1997 ▶); program(s) used to solve structure: *SHELXS97* (Sheldrick, 2008 ▶); program(s) used to refine structure: *SHELXL97* (Sheldrick, 2008 ▶); molecular graphics: *PLATON* (Spek, 2009 ▶); software used to prepare material for publication: *SHELXL97*.

## Supplementary Material

Crystal structure: contains datablocks I, global. DOI: 10.1107/S160053680900988X/bx2197sup1.cif

Structure factors: contains datablocks I. DOI: 10.1107/S160053680900988X/bx2197Isup2.hkl

Additional supplementary materials:  crystallographic information; 3D view; checkCIF report

**Fig. 1.**
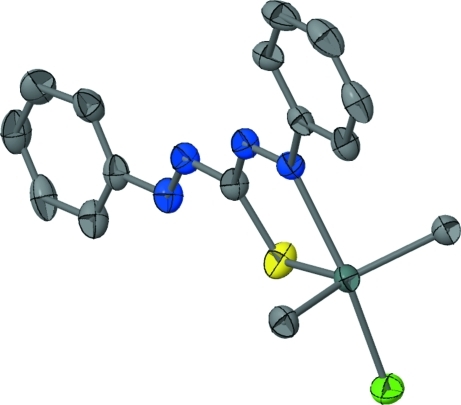
The molecular structure of (I), with the atom-numbering scheme. Displacement ellipsoids are drawn at the 50% probability level. H atoms have been omitted.

Enhanced figure: interactive version of Fig. 1

## supplementary crystallographic information

### Comment

The interest in the synthesis of new organotin derivatives is related to the diversity of structures that such compounds can form, and on the other side, to their biological and pharmaceutical applications (Davies & Smith, 1982,Okio *et al.*, 2003; Diop *et al.*, 2003). We report here the crystal structure of the title compound (I). The structure consists of discrete molecules (Fig.1).The Sn atom is five-coordinate in a distorted trigonal–bipyramidal geometry. The distorted trigonal-bipyramidal coordination polyhedron has two methyl groups and one S atom in the equatorial plane, the N1 and Cl atom occupying the apical positions. The Sn—Cl and Sn—S bond distances are shorter than the  values of  2.672 (1) Å and 2.478 (2) Å found in dimethylmonochloro[(*N*-(2-pyridinylmethylene) hydrazinecarbothioamidato)NS(– 1)]tin(IV) hemihydrate ( Labib *et al.*,1996), while the Sn—N and Sn—C bonds are longer than the corresponding ones reported for the same previous compound, (2.359 (4) Å, 2.108 (7) Å and 2.105 (7) Å), as representative example.

### Experimental

Compound (I) was obtained by reacting dimethyltin (IV) dichloride (220 mg, 1 mmol) with 1,5 diphenylthiocarbazone (128 mg, 0.5 mmol) in dichloromethane under reflux for 3 h. Dark red crystals suitable for X-ray analysis were grown by slow solvent evaporation.

### Refinement

H atoms were positioned geometrically, with C—H distances in the range 0.95 - 0.98 Å, and constrained to ride on their parent atoms, with *U*_iso_(H) = 1.2-1.5U_eq_(C). H atom bonded to N4 was found in difference maps and positioned geometrically (N4-H = 0.88 Å) and was refined with a riding model, with U_iso_(H) = 1.2U_eq_(C).

### Crystal data

**Table:**

[Sn(CH_3_)_2_(C_13_H_11_N_4_S)Cl]	*F*(000) = 1744
*M**_r_* = 439.53	*D*_x_ = 1.623 Mg m^−^^3^
Orthorhombic, *P**b**n**a*	Mo *K*α radiation, λ = 0.71073 Å
Hall symbol: -P 2ac 2b	Cell parameters from 14356 reflections
*a* = 11.6850 (2) Å	θ = 1.0–27.5°
*b* = 14.8920 (5) Å	µ = 1.69 mm^−^^1^
*c* = 20.6790 (7) Å	*T* = 173 K
*V* = 3598.42 (18) Å^3^	Prism, dark red
*Z* = 8	0.10 × 0.10 × 0.10 mm

### Data collection

**Table:**

Nonius KappaCCD diffractometer	2731 reflections with *I* > 2σ(*I*)
Radiation source: fine-focus sealed tube	*R*_int_ = 0.115
graphite	θ_max_ = 27.5°, θ_min_ = 2.0°
π scans	*h* = −15→15
25644 measured reflections	*k* = −18→19
4130 independent reflections	*l* = −26→22

### Refinement

**Table:**

Refinement on *F*^2^	Primary atom site location: structure-invariant direct methods
Least-squares matrix: full	Secondary atom site location: difference Fourier map
*R*[*F*^2^ > 2σ(*F*^2^)] = 0.044	Hydrogen site location: inferred from neighbouring sites
*wR*(*F*^2^) = 0.137	H-atom parameters constrained
*S* = 1.09	*w* = 1/[σ^2^(*F*_o_^2^) + (0.07*P*)^2^] where *P* = (*F*_o_^2^ + 2*F*_c_^2^)/3
4130 reflections	(Δ/σ)_max_ = 0.002
199 parameters	Δρ_max_ = 1.13 e Å^−^^3^
0 restraints	Δρ_min_ = −1.84 e Å^−^^3^

### Special details

**Table:**

Geometry. All e.s.d.'s (except the e.s.d. in the dihedral angle between two l.s. planes) are estimated using the full covariance matrix. The cell e.s.d.'s are taken into account individually in the estimation of e.s.d.'s in distances, angles and torsion angles; correlations between e.s.d.'s in cell parameters are only used when they are defined by crystal symmetry. An approximate (isotropic) treatment of cell e.s.d.'s is used for estimating e.s.d.'s involving l.s. planes.
Refinement. Refinement of *F*^2^ against ALL reflections. The weighted *R*-factor *wR* and goodness of fit *S* are based on *F*^2^, conventional *R*-factors *R* are based on *F*, with *F* set to zero for negative *F*^2^. The threshold expression of *F*^2^ > σ(*F*^2^) is used only for calculating *R*-factors(gt) *etc*. and is not relevant to the choice of reflections for refinement. *R*-factors based on *F*^2^ are statistically about twice as large as those based on *F*, and *R*- factors based on ALL data will be even larger.

### Fractional atomic coordinates and isotropic or equivalent isotropic displacement parameters (Å^2^)

**Table:**

	*x*	*y*	*z*	*U*_iso_*/*U*_eq_	
Sn	0.18590 (3)	0.56103 (2)	0.509606 (17)	0.03083 (14)	
Cl	0.03058 (11)	0.57242 (10)	0.59081 (6)	0.0489 (4)	
S	0.30578 (10)	0.64147 (10)	0.58678 (6)	0.0440 (4)	
N1	0.3690 (3)	0.5795 (2)	0.45454 (18)	0.0308 (9)	
N2	0.4565 (3)	0.6110 (3)	0.48494 (17)	0.0325 (9)	
N3	0.5342 (3)	0.6667 (2)	0.57432 (18)	0.0343 (9)	
N4	0.5270 (3)	0.6917 (2)	0.63558 (19)	0.0364 (9)	
H4N	0.4606	0.6889	0.6556	0.044*	
C1	0.3037 (4)	0.5110 (3)	0.3553 (2)	0.0368 (11)	
H1	0.2316	0.5011	0.3754	0.044*	
C2	0.3220 (4)	0.4841 (3)	0.2918 (3)	0.0444 (13)	
H2	0.2620	0.4559	0.2683	0.053*	
C3	0.4263 (5)	0.4979 (3)	0.2626 (2)	0.0516 (14)	
H3	0.4380	0.4797	0.2191	0.062*	
C4	0.5137 (5)	0.5383 (4)	0.2966 (3)	0.0484 (14)	
H4	0.5856	0.5474	0.2761	0.058*	
C5	0.4985 (4)	0.5659 (3)	0.3598 (2)	0.0364 (11)	
H5	0.5595	0.5934	0.3829	0.044*	
C6	0.3923 (4)	0.5527 (3)	0.3896 (2)	0.0316 (10)	
C7	0.6144 (4)	0.7333 (3)	0.7357 (2)	0.0423 (13)	
H7	0.5451	0.7188	0.7573	0.051*	
C8	0.7073 (5)	0.7652 (3)	0.7698 (3)	0.0474 (14)	
H8	0.7011	0.7735	0.8152	0.057*	
C9	0.8086 (4)	0.7852 (4)	0.7392 (3)	0.0503 (14)	
H9	0.8718	0.8075	0.7633	0.060*	
C10	0.8178 (4)	0.7729 (4)	0.6738 (3)	0.0518 (15)	
H10	0.8884	0.7856	0.6529	0.062*	
C11	0.7263 (5)	0.7424 (3)	0.6376 (3)	0.0425 (13)	
H11	0.7330	0.7351	0.5921	0.051*	
C12	0.6243 (4)	0.7226 (3)	0.6692 (2)	0.0342 (11)	
C13	0.4384 (4)	0.6391 (3)	0.5470 (2)	0.0329 (10)	
C14	0.2130 (5)	0.4204 (4)	0.5157 (3)	0.0438 (14)	
H14A	0.1517	0.3890	0.4926	0.066*	
H14B	0.2129	0.4019	0.5612	0.066*	
H14C	0.2869	0.4053	0.4961	0.066*	
C15	0.1051 (3)	0.6462 (3)	0.4410 (2)	0.0374 (11)	
H15A	0.0860	0.6115	0.4022	0.056*	
H15B	0.1572	0.6952	0.4293	0.056*	
H15C	0.0350	0.6712	0.4598	0.056*	

### Atomic displacement parameters (Å^2^)

**Table:**

	*U*^11^	*U*^22^	*U*^33^	*U*^12^	*U*^13^	*U*^23^
Sn	0.0292 (2)	0.0351 (2)	0.0282 (2)	0.00016 (13)	−0.00196 (12)	0.00342 (13)
Cl	0.0390 (7)	0.0750 (10)	0.0326 (7)	0.0030 (6)	0.0051 (5)	0.0065 (6)
S	0.0344 (6)	0.0634 (9)	0.0342 (8)	0.0036 (6)	−0.0021 (5)	−0.0164 (6)
N1	0.033 (2)	0.029 (2)	0.031 (2)	−0.0025 (16)	−0.0031 (17)	0.0010 (16)
N2	0.035 (2)	0.030 (2)	0.032 (2)	−0.0017 (18)	−0.0044 (17)	−0.0016 (17)
N3	0.037 (2)	0.033 (2)	0.033 (2)	−0.0034 (16)	−0.0054 (17)	−0.0021 (18)
N4	0.039 (2)	0.037 (2)	0.034 (2)	0.0020 (18)	−0.0062 (17)	−0.0077 (18)
C1	0.044 (3)	0.035 (3)	0.032 (3)	0.002 (2)	−0.007 (2)	−0.001 (2)
C2	0.069 (4)	0.031 (3)	0.033 (3)	0.008 (3)	−0.017 (3)	−0.006 (2)
C3	0.082 (4)	0.042 (3)	0.031 (3)	0.013 (3)	0.000 (3)	−0.001 (2)
C4	0.066 (3)	0.043 (3)	0.036 (3)	0.011 (3)	0.018 (3)	0.006 (2)
C5	0.043 (3)	0.033 (3)	0.033 (3)	0.002 (2)	0.005 (2)	0.002 (2)
C6	0.040 (2)	0.027 (3)	0.028 (3)	0.0068 (19)	−0.002 (2)	0.0025 (19)
C7	0.045 (3)	0.046 (3)	0.036 (3)	0.012 (2)	−0.007 (2)	−0.009 (2)
C8	0.061 (3)	0.041 (3)	0.040 (3)	0.019 (2)	−0.021 (3)	−0.012 (2)
C9	0.050 (3)	0.041 (3)	0.060 (4)	−0.001 (3)	−0.024 (3)	−0.007 (3)
C10	0.048 (3)	0.048 (3)	0.060 (4)	−0.018 (2)	−0.013 (3)	0.006 (3)
C11	0.049 (3)	0.038 (3)	0.040 (3)	−0.007 (2)	−0.012 (2)	0.005 (2)
C12	0.037 (3)	0.025 (2)	0.040 (3)	0.004 (2)	−0.010 (2)	−0.004 (2)
C13	0.035 (2)	0.032 (3)	0.032 (3)	0.000 (2)	−0.004 (2)	−0.004 (2)
C14	0.042 (3)	0.037 (3)	0.052 (4)	0.003 (2)	−0.002 (2)	0.010 (2)
C15	0.036 (2)	0.038 (3)	0.038 (3)	0.000 (2)	−0.003 (2)	0.006 (2)

### Geometric parameters (Å, °)

**Table:**

Sn—C14	2.122 (5)	C4—H4	0.9500
Sn—C15	2.125 (5)	C5—C6	1.399 (6)
Sn—S	2.4380 (13)	C5—H5	0.9500
Sn—N1	2.439 (4)	C7—C8	1.379 (7)
Sn—Cl	2.4784 (13)	C7—C12	1.388 (7)
S—C13	1.755 (4)	C7—H7	0.9500
N1—N2	1.289 (5)	C8—C9	1.375 (8)
N1—C6	1.428 (6)	C8—H8	0.9500
N2—C13	1.366 (6)	C9—C10	1.370 (8)
N3—C13	1.320 (5)	C9—H9	0.9500
N3—N4	1.323 (5)	C10—C11	1.382 (7)
N4—C12	1.410 (6)	C10—H10	0.9500
N4—H4N	0.8800	C11—C12	1.392 (7)
C1—C2	1.390 (7)	C11—H11	0.9500
C1—C6	1.400 (6)	C14—H14A	0.9800
C1—H1	0.9500	C14—H14B	0.9800
C2—C3	1.375 (7)	C14—H14C	0.9800
C2—H2	0.9500	C15—H15A	0.9800
C3—C4	1.377 (7)	C15—H15B	0.9800
C3—H3	0.9500	C15—H15C	0.9800
C4—C5	1.383 (7)		
			
C14—Sn—C15	134.0 (2)	C5—C6—N1	122.9 (4)
C14—Sn—S	111.14 (15)	C1—C6—N1	117.3 (4)
C15—Sn—S	113.52 (14)	C8—C7—C12	118.8 (5)
C14—Sn—N1	90.45 (17)	C8—C7—H7	120.6
C15—Sn—N1	90.60 (14)	C12—C7—H7	120.6
S—Sn—N1	75.30 (9)	C9—C8—C7	121.1 (5)
C14—Sn—Cl	97.86 (15)	C9—C8—H8	119.4
C15—Sn—Cl	94.95 (13)	C7—C8—H8	119.4
S—Sn—Cl	86.77 (4)	C10—C9—C8	119.5 (5)
N1—Sn—Cl	161.98 (9)	C10—C9—H9	120.2
C13—S—Sn	100.99 (16)	C8—C9—H9	120.2
N2—N1—C6	114.2 (4)	C9—C10—C11	121.3 (5)
N2—N1—Sn	120.6 (3)	C9—C10—H10	119.4
C6—N1—Sn	125.1 (3)	C11—C10—H10	119.4
N1—N2—C13	116.6 (4)	C10—C11—C12	118.5 (5)
C13—N3—N4	116.3 (4)	C10—C11—H11	120.7
N3—N4—C12	120.8 (4)	C12—C11—H11	120.7
N3—N4—H4N	119.6	C7—C12—C11	120.8 (4)
C12—N4—H4N	119.6	C7—C12—N4	117.3 (4)
C2—C1—C6	119.5 (4)	C11—C12—N4	121.9 (4)
C2—C1—H1	120.3	N3—C13—N2	111.5 (4)
C6—C1—H1	120.3	N3—C13—S	122.8 (4)
C3—C2—C1	120.5 (5)	N2—C13—S	125.7 (3)
C3—C2—H2	119.7	Sn—C14—H14A	109.5
C1—C2—H2	119.7	Sn—C14—H14B	109.5
C2—C3—C4	120.0 (5)	H14A—C14—H14B	109.5
C2—C3—H3	120.0	Sn—C14—H14C	109.5
C4—C3—H3	120.0	H14A—C14—H14C	109.5
C3—C4—C5	121.1 (5)	H14B—C14—H14C	109.5
C3—C4—H4	119.4	Sn—C15—H15A	109.5
C5—C4—H4	119.4	Sn—C15—H15B	109.5
C4—C5—C6	119.2 (5)	H15A—C15—H15B	109.5
C4—C5—H5	120.4	Sn—C15—H15C	109.5
C6—C5—H5	120.4	H15A—C15—H15C	109.5
C5—C6—C1	119.8 (4)	H15B—C15—H15C	109.5
			
C14—Sn—S—C13	77.8 (2)	C2—C1—C6—N1	179.6 (4)
C15—Sn—S—C13	−91.0 (2)	N2—N1—C6—C5	−4.7 (6)
N1—Sn—S—C13	−6.86 (18)	Sn—N1—C6—C5	179.1 (3)
Cl—Sn—S—C13	175.02 (17)	N2—N1—C6—C1	174.9 (4)
C14—Sn—N1—N2	−104.0 (3)	Sn—N1—C6—C1	−1.3 (5)
C15—Sn—N1—N2	121.9 (3)	C12—C7—C8—C9	−0.8 (7)
S—Sn—N1—N2	7.7 (3)	C7—C8—C9—C10	−0.3 (8)
Cl—Sn—N1—N2	13.8 (5)	C8—C9—C10—C11	1.3 (9)
C14—Sn—N1—C6	71.9 (3)	C9—C10—C11—C12	−1.2 (8)
C15—Sn—N1—C6	−62.1 (3)	C8—C7—C12—C11	1.0 (7)
S—Sn—N1—C6	−176.3 (3)	C8—C7—C12—N4	−178.6 (4)
Cl—Sn—N1—C6	−170.2 (2)	C10—C11—C12—C7	0.0 (7)
C6—N1—N2—C13	179.0 (4)	C10—C11—C12—N4	179.6 (5)
Sn—N1—N2—C13	−4.6 (5)	N3—N4—C12—C7	−169.6 (4)
C13—N3—N4—C12	−179.8 (4)	N3—N4—C12—C11	10.9 (7)
C6—C1—C2—C3	0.2 (7)	N4—N3—C13—N2	−177.1 (4)
C1—C2—C3—C4	0.3 (8)	N4—N3—C13—S	4.1 (6)
C2—C3—C4—C5	−0.3 (8)	N1—N2—C13—N3	177.5 (4)
C3—C4—C5—C6	−0.3 (7)	N1—N2—C13—S	−3.8 (6)
C4—C5—C6—C1	0.8 (7)	Sn—S—C13—N3	−172.3 (4)
C4—C5—C6—N1	−179.6 (4)	Sn—S—C13—N2	9.1 (4)
C2—C1—C6—C5	−0.8 (7)		
